# Potential Seasonal Terrestrial Water Storage Monitoring from GPS Vertical Displacements: A Case Study in the Lower Three-Rivers Headwater Region, China

**DOI:** 10.3390/s16091526

**Published:** 2016-09-19

**Authors:** Bao Zhang, Yibin Yao, Hok Sum Fok, Yufeng Hu, Qiang Chen

**Affiliations:** 1 School of Geodesy and Geomatics, Wuhan University, Wuhan 430079, China; sggzb@whu.edu.cn (B.Z.); yfhu@whu.edu.cn (Y.H.); 2 Faculte des Science, de la Technologie et de la Communication, University of Luxembourg, 6 Rue Richard Coudenhove-Kalergi L-1359, Luxembourg; qiang.chen@uni.lu

**Keywords:** GPS, terrestrial water storage, GRACE, GLDAS, three-rivers

## Abstract

This study uses the observed vertical displacements of Global Positioning System (GPS) time series obtained from the Crustal Movement Observation Network of China (CMONOC) with careful pre- and post-processing to estimate the seasonal crustal deformation in response to the hydrological loading in lower three-rivers headwater region of southwest China, followed by inferring the annual EWH changes through geodetic inversion methods. The Helmert Variance Component Estimation (HVCE) and the Minimum Mean Square Error (MMSE) criterion were successfully employed. The GPS inferred EWH changes agree well qualitatively with the Gravity Recovery and Climate Experiment (GRACE)-inferred and the Global Land Data Assimilation System (GLDAS)-inferred EWH changes, with a discrepancy of 3.2–3.9 cm and 4.8–5.2 cm, respectively. In the research areas, the EWH changes in the Lancang basin is larger than in the other regions, with a maximum of 21.8–24.7 cm and a minimum of 3.1–6.9 cm.

## 1. Introduction

The three-rivers headwater region, known as “China’s Water Tower” located in the eastern Qinghai-Tibet Plateau, represents a fundamental water supply region for the Lancang River, Yangtze River, and Yellow River in China that is sensitive to global climate change [1]. With the global warming, the shrinkage of lakes size, the decline in runoff flow, and the degradation of grassland, are but a few scenarios consequently posing an adverse effect on the middle and the lower river basins in China and in East Asian countries [2]. Therefore, large-scale quantification and monitoring is essential for resolving the challenges and conflicts of water supply at both national and international level.

Recently, space-geodetic observations have been widely used to infer terrestrial water storage (TWS) at both a global and regional scale, due to spatially sparse ground-based in-situ observations and insufficient fund to maintain a number of hydrologic monitoring stations. GRACE is one of the space-geodetic missions for monitoring time-variable gravity changes from space, and hence, providing an opportunity to detect the surface mass changes [3], in which water movement cycle is a substantial component [4]. GRACE derived TWS has been compared with the estimates from GLDAS NOAH hydrologic model data (e.g., [5,6]). Though GRACE is capable of detecting TWS change with some accuracy for regions that are several hundred km or more in scale, it has a low spatial resolution (~300 km) which has limited its application in small regions [5]. Hydrologic models, like GLDAS, contain only soil moisture, snow, and plant canopy surface water, and they can’t reflect the total TWS change, especially water in the river, reservoir and underground. GPS coordinates (especially in up components) are more sensitive to surface mass change than GRACE, which providing a potential way to detect TWS change with higher resolution. Since this century, seasonal deformation due to surface water transport has been detected by GPS (e.g., [7,8,9,10]), and by the combination of GPS and GRACE (e.g., [11,12,13,14,15]). More recently, Steckler et al. [16] applied GPS, and GRACE data to model the Earth deformation caused by monsoonal flooding in Bangladesh validated with water level in river gauge stations. Chew and Small [17] estimated the TWS response to drought in year 2012 from the GPS vertical position anomalies in central United States. Birhanu and Bendick [18] used the GPS displacement time series to investigate the hydrological loading caused by precipitation in Ethiopia and Eritrea. All the aforementioned research studies convert GRACE gravity field to vertical deformation comparable to that measured by GPS.

The technique for obtaining the change in water thickness from GPS vertical displacements through a well-known Green’s function [19], has been newly developed to investigate the TWS in California. The obtained TWS is comparable to that from GRACE and hydrologic models [20]. These pioneer studies have been making an advancement on the application of GPS to hydrology. Seasonal vertical oscillations of 922 GPS sites are successfully used to invert the seasonal variation in total water storage in California [20] which are also compared with GRACE results and hydrological models.

The CMONOC network, with access to more than 200 continuous stations since 2009, is a network for monitoring the crustal deformation, with stations less dense when compared to the Plate Boundary Observatory (PBO) network in United States. This study aims to investigate the potential usage of CMONOC GPS stations for inverting the seasonal variation of TWS in Yunnan Province in China, where it is connected to Lancang River (also called Mekong River system outside the boundaries of China), with the Yangtze River and Zhujiang River nearby. Its annual total precipitation can reach more than 1000 mm, but ~83% of that happened from May to October and the other 17% happened from November to next April. The seasonal change of precipitation in this region is so large, which will enlarge the seasonal signal of TWS, making it a good experimental region for this study. Two algorithms are employed to obtain the resulting EWH, followed by an accuracy assessment and validation with GPS and GRACE and GLDAS NOAH hydrologic model.

## 2. GPS Data Analysis

34 GPS sites from the CMONOC network are located within the region from 21.5°N to 29.5°N and from 97.5°E to 105.5°E near the Lancang River, Yangtze River and Zhujiang River. Among the 34 sites, the XANG and KMIN started operating in 2000 and 2004, respectively, and the other 32 sites started operating in 2011. GPS observation data from the start operating time to 2014 are processed by the GAMIT/GLOBK [21] to produce precise site coordinates. The data pre-processing and post-processing steps are detailed in the below sub-sections.

### 2.1. Data Pre-Processing with GAMIT/GLOBK

The GAMIT software incorporates a weighted least squares algorithm to estimate relative positions of a set of stations, orbital and Earth-rotation parameters, tropospheric delays, and phase ambiguities by fitting to double-differenced phase observations. To generate absolute coordinate solutions in the International Terrestrial Reference Frame (ITRF2008) [22], GPS observation data from 24 International GNSS Service (IGS) [23] stations were processed together with the 34 CMONOC stations. The uncertainties of the IGS station coordinates were constrained to 5 cm by using coordinates (in itrf2008.apr file) from MIT, whereas that of the other 34 sites were initially constrained to 100 m by using coordinates from point-position solution.

Standard corrections have been applied in the pre-processing. The satellites orbits are fixed by using IGS final orbit products. The first-order ionospheric delay is eliminated by ionosphere-free linear combination, while the second and third order terms are corrected by models [24] in Gamit 10.4 [25]. The tropospheric delay was modeled as piecewise linear model and estimated. The a priori hydrostatic delay and mapping functions could influence the accuracy of the coordinates and tropospheric delay estimation. So the tropospheric delay effects should be carefully dealt with when generating GPS position time series. As the Vienna Mapping Function 1 (VMF1 [26]) provides slightly better station height repeatabilities (generally within ±1 mm difference) than Global Mapping Function (GMF [27]) [28], so VMF1 is employed to provide mapping functions with a cut-off value of 10°. As no accurate site-dependent hydrostatic delays available, we use the Global Pressure and Temperature (GPT) model [29] to provide a priori hydrostatic delays. Non-tidal atmospheric loading and ocean tidal loading are corrected by MIT atmdisp_cm.year files and FES2004 model in GAMIT, respectively. The International Earth Rotation Service (IERS) 2003 model for diurnal and semi-diurnal solid Earth tides was set. The pole tide was also corrected in GAMIT by IERS standards. The satellite and receiver antenna center offsets are corrected by using the IGS antenna correction files. The Earth Orientation Parameters (EOP) were constrained tightly to priori values obtained from IERS Bulletin B. By these settings, the GAMIT were used to process the GPS data from the total 58 sites to produce baseline solutions with their uncertainties. Then the script glred in GLOBK are used to process the GAMIT results and generates daily coordinate solutions and their uncertainties. As the coordinates of the 24 IGS stations were tightly constrained, the final coordinate solutions were tied to ITRF2008.

Previous studies have stated the significance of environmental loading (i.e., loading contributions due to changes in atmospheric loading (ATML), continental water storage (CWS), and non-tidal ocean loading (NTOL) [30]. When processing the GPS observation data, the ATML contributions has been corrected but taking no account for CWS and NTOL. As a result, correction for the vertical displacements induced by NTOL should be taken into consideration, and thus, obtaining the signal caused by CWS loadings. The displacements induced from NTOL are corrected by using the Global Geophysical Fluid Center (GGFC) 12-hourly, global surface displacements with 2.5° × 2.5° spatial resolution. This data set is derived from the estimating the circulation and climate of the ocean (ECCO) global model. Using the Fortran code *ntol_tseries.f* provided by ggfc-oceans, NTOL correction can be extracted for a specified latitude and longitude. As the GPS sites are far away from the sea (the nearest site YNJP is about 450 km away from the coast), so the NTOL has very slight influence on the sites vertical positions (no more than 0.5 mm on the annual amplitude estimation).

### 2.2. Post-Processing of Coordinates Time Series

After the pre-processing steps, 58 vertical coordinate time series were obtained, among which 34 were located in the research area. Post-processing is essential to scrutinizing and enhancing data quality. Criteria are required and described in the following sub-sections.

#### 2.2.1. Outlier Rejection of GPS Data

**Figure 1:**
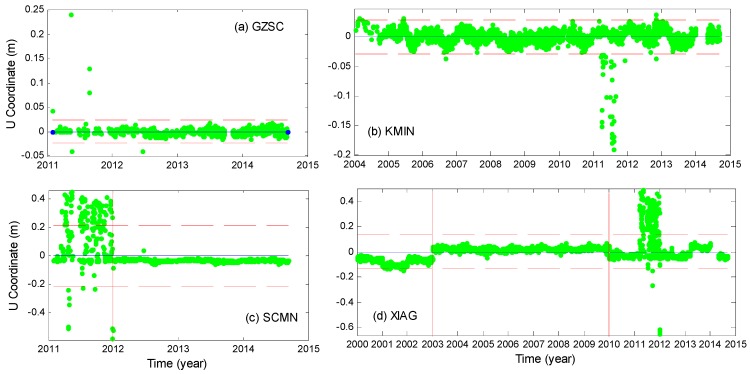
(**a**–**d**) Daily values of vertical relative positions (with linear term removal) at four typical stations, with the mean position (blue solid lines), 2σ range (red dash lines), and the abrupt changes (red solid lines) when jumps are presented.

As the data quality of CMONOC are not as good as that of the IGS stations, especially in 2011 when ionospheric anomalies frequently happened, extra steps are designed to remove data outliers and to ensure the quality of the final estimates. The presence of outliers can be due to earthquake occurrence, the antenna changes, the occasional time series discontinuities and bad data quality. Therefore, the time series were sub-divided into four groups, according to the outlier characteristics of the time series at 34 stations. The four types of time series (Figure 1) are classified as follows: (a): most of the relative positions fall within 2σ (standard deviation) range and no antenna changes (e.g., GZSC); (b): most of the relative positions fall within 2σ range with some position jumps and no antenna change (e.g., KMIN); (c): the relative positions are highly scattered in the first year but stabilized after year 2012 (e.g., SCMN); (d): the relative positions jumped due to obvious antenna movement (e.g., XIAG).

These kinds of coordinate time series are not qualified for estimating the TWS, extra steps are required to remove the outliers.

Step 1: Calculate the mean position and standard deviation and remove data that are beyond the range of ±2σ;
Step 2: Find those stations belong to Group (c) and remove data before year 2012;
Step 3: Find those stations belong to Group (d) and estimate the antenna offset; in fact, antenna offset is still present in XIAG station after Step 2. Hence, data before 2003 and after 2010 were removed.
Step 4: Redo Step 1.

After the above steps, large gross errors are eliminated and relatively clean data are generated within the 2σ range with no obvious outliers.

#### 2.2.2. Regional Filtering of the GPS Data

**Figure 2:**
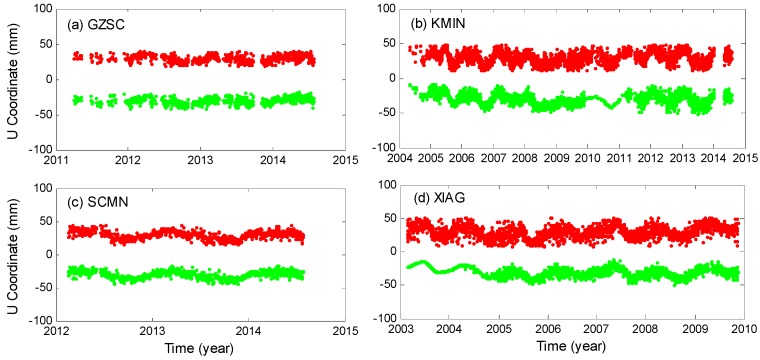
(**a**–**d**) Time series of vertical positions at four stations before (red dots) and after (green dots) regional filtering. The time series are shifted by an offset for clarity.

To reduce the spatially-correlated (or regional common modes) noise in the GPS time series that potentially arise from tropospheric modeling, satellite orbit configuration, and related errors, the ‘regional filter’ [31,32] is employed on the GPS data after the gross errors are removed. The time series before and after regional filtering are illustrated in Figure 2. A reduction of the data scatter is observed after the regional filtering, but the linear, annual and inter-annual signals are preserved. Note that subtle fluctuations occurred between 2010–2011 at KMIN and between 2003–2005 at XIAG due to the fact that only one station’s data are available during these time, so when doing the ‘regional filter’, we actually just remove the high-frequency components of the time series at these two stations, which is not exactly a ‘regional filter’, so data during these periods at these two sites will not be used.

#### 2.2.3. Validation of Seasonal Signals

**Figure 3:**
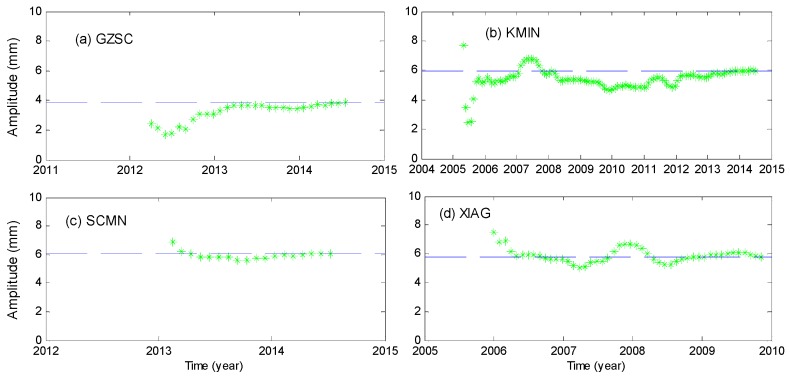
(**a**–**d**) Convergence plots of annual amplitude at GZSC, KMIN, SCMN, XIAG. Obtained by fitting constant, linear and annual terms to the first 365 days of the data, followed by adding that of the next 30 days and the fit is repeated until all data are used.

To yield a true and a stable seasonal signal from GPS time series, assessment of the reliability of the estimated amplitudes is required. To achieve this purpose, a model with a constant, a linear and an annually varying term are fitted to the GPS time series at each site. To be precise, we first use data of the first 365 days to fit the temporal model, followed by adding the next 30-day data (for the fitting results see Figure 3). The fitting procedures are repeated until the whole data time series are used up. If the difference between the amplitude estimated using data before the last year and the final amplitude using full data is within 1 mm, the estimated amplitude is believed to be reliable. By this criterion, station YNYL is excluded.

At station XIAG, the amplitude before 2005.5 is not consistent with that after 2005.5 due to the incorrect regional filtering (before 2005 only XIAG station’s data are available), so we delete the data before 2005 at XIAG. Figure 3 shows the convergence plots for GPS vertical annual amplitudes. The signal-to-noise ratio (i.e., the annual amplitude to the Root Mean Square Error (RMSE) of the post fit residuals) served as another indicator for rejection. If the ratio is larger than 1, the resulting amplitudes are accepted. By this criterion, the stations KMIN, YNGM, YNWS, YNXP are excluded. Finally, only 29 stations are left and used.

### 2.3. Annual Amplitude Estimation

**Figure 4:**
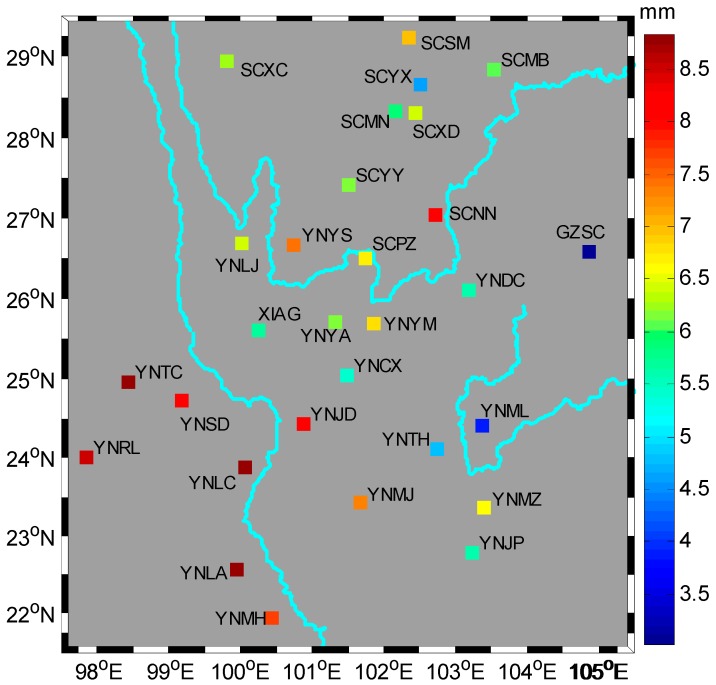
Annual amplitudes of the vertical positions at the 29 stations estimated by maximum likelihood method, the noise model is power law plus white noise.

To estimate accurate linear or seasonal parameters and realistic uncertainties from GPS time series, it is important to understand the noise contents in GPS time series. Both Zhang et al. [33] and Mao et al. [34] concluded that the uncertainties will be grossly underestimated when only white noise model is considered. As with other geophysical phenomena, noise in GPS position time series can be described as a power law process [35,36]. Flicker noise and random walk noise are two canonical types of noise models, which have been confirmed to be present in GPS time series [37]. To obtain accurate parameters and their uncertainties, here we don’t constrain the colored noise to flicker noise or random walk noise but prefer a power law noise plus white noise model to describe the stochastic process. The Hector program [38] is used to estimate the bias, trend, annual and semi-annual terms, together with the power law noise and its spectral index and the white noise by maximum likelihood estimation. The chosen method for the likelihood computation is the superfast solution proposed by Ammar and Gragg [39]. Figure 4 shows the locations and amplitudes of 29 stations while Table 1 shows the noise components, spectral indexes and annual amplitudes and the uncertainties. According to the statistics in Table 1, the mean white noise and power law noise of the GPS time series are 3.28 mm and 9.60 mm/year^1/4^, respectively. The mean spectral index is −0.86 which is comparable to the results (−0.89 ± 0.28) from Mao et al. [34]. The mean amplitude of annual oscillation induced by seasonal TWS change in the research area is about 6.59 mm.

## 3. Determination of Water Thickness from GPS

**Figure 5:**
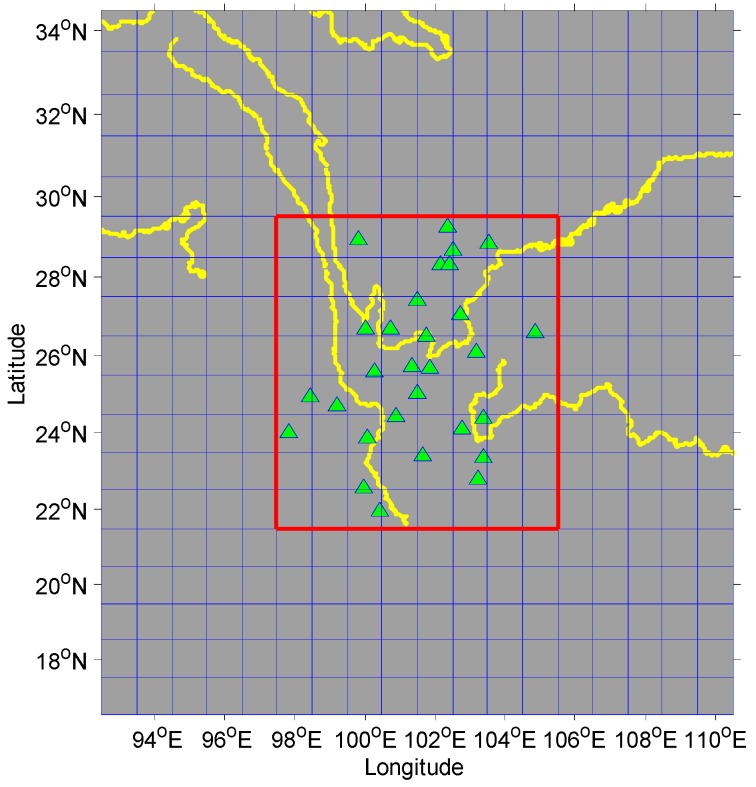
Plots of the grid cells (with 1° × 1° size) and GPS station locations. The core area is inside the red box and the outside is the additional area.

Since the limited number of GPS stations within the study region, the annual amplitude (i.e., seasonal vertical oscillations) cannot be inverted to infer EWH at a high spatial resolution. Thus, the study region (latitude: 21.5°N–29.5°N, longitude: 97.5°E–105°E) was subdivided into 1° × 1° grids. To take into account the hydrologic loading outside the region, the region is extended by an extra 5° (Figure 5), where the estimation of EWH in the core areas are of fundamental interest.

In this set, we have 324 EWH parameters (64 in the core area) to be estimated, with only 29 annual amplitudes of the GPS vertical positions as observations. By using the Green’s functions [19], one can relate the elastic response (i.e., GPS vertical observations) to Earth surface loading (i.e., the unknown EWH parameters). For a point load, the vertical displacement it caused can be expressed as:

(1) u=G(θ)×m

where *u* is the vertical displacement (in meters), *m* is the mass of load (in kilograms) and *G* is the Green’s function of θ, the angular distance between the point load and the station. Theoretically, we should integrate vertical displacements caused by all point loads on the Earth surface to equal the total vertical displacement of the station, but this is impractical. In fact, the elastic vertical motion decreases rapidly with the distance from a load.

A large integrating area means more unknown parameters to be estimated that makes the ill-posed problem even worse. Thus, the additional area with a width of 5° is defined so that hydrological loads in the additional and core area are considered together. Since the number of unknown EWH parameters are larger than GPS observations, additional constraints are required to invert the EWH parameters. Here, the Laplacian operator, *B*, are served as the additional constraints that smooth the solution. The equations are as follows:

(2) $$\begin{array}{l} L=AX\\ 0=BX \end{array}\}$$

where $A=\left[\begin{array}{cccc} \sum_{\phi ,\lambda }^{\Omega_{1}^{1}}\rho G\cdot dS & \sum_{\phi ,\lambda }^{\Omega_{2}^{1}}\rho G\cdot dS & \cdots & \sum_{\phi ,\lambda }^{\Omega_{324}^{1}}\rho G\cdot dS\\ \sum_{\phi ,\lambda }^{\Omega_{1}^{2}}\rho G\cdot dS & \sum_{\phi ,\lambda }^{\Omega_{2}^{2}}\rho G\cdot dS & \cdots & \sum_{\phi ,\lambda }^{\Omega_{324}^{2}}\rho G\cdot dS\\ \vdots & \vdots & \ddots & \vdots \\ \sum_{\phi ,\lambda }^{\Omega_{1}^{29}}\rho G\cdot dS & \sum_{\phi ,\lambda }^{\Omega_{2}^{29}}\rho G\cdot dS & \cdots & \sum_{\phi ,\lambda }^{\Omega_{324}^{29}}\rho G\cdot dS \end{array}\right]$, $L=\left[\begin{array}{c} u_{1}\\ u_{2}\\ \vdots \\ u_{29} \end{array}\right]$, $X=\left[\begin{array}{c} H_{1}\\ H_{2}\\ \vdots \\ H_{324} \end{array}\right]$.

where *A* is the design matrix of observation equations consisting of integrated vertical motion caused by 1 m height water change. ϕ and λ are latitude and longitude. During integration, each 0.025° × 0.025° area is treated as a point load. *L* is the observations consisting of the annual amplitudes of the vertical positions at the 29 sites. *X* is the vector of the 324 EWH parameters. *ρ* is the density of liquid water, *dS* is the area of the integral element. *G* is Green’s functions which are consistent with those calculated by Guo et al. [41] for the preliminary reference Earth model (PREM) of Dziewonski and Anderson [42]. The solution for Equation (2) can be written as:

(3) $$\hat{X}={\left(A^{T}PA+kB^{T}B\right)}^{-1}A^{T}PL$$

where *P* is the weight matrix of *L*, *k* is an unknown nonnegative parameter. This becomes the classic regularization problem. If *k* is set to zero, $\hat{X}$ is the least squares (LS) solution, but here we cannot get a reliable LS solution. The regularized solution (3) depends on the choice of a proper regularization parameter *k*. Many methods have been developed to determine the *k*, including L-curve method [43,44,45], MMSE criterion method [46], the principal components estimator [47], Akaike’s Bayesian information criterion [48] and the HVCE method [49,50], etc. Here, we choose the MMSE and HVCE methods to determine *k*, respectively. The MMSE method is performed using the same procedures as in [46] and will not be further illustrated here. The equations for HVCE in this study can be written as:

(4) $$S\hat{\theta }=W$$

where $S=\left[\begin{array}{cc} n_{1}-2tr(N^{-1}N_{1})+tr(N^{-1}N_{1}N^{-1}N_{1}) & tr(N^{-1}N_{1}N^{-1}N_{2})\\ tr(N^{-1}N_{1}N^{-1}N_{2}) & n_{2}-2tr(N^{-1}N_{2})+tr(N^{-1}N_{2}N^{-1}N_{2}) \end{array}\right]$, $\hat{\theta }={\left[\begin{array}{cc} {\hat{\sigma }}_{01}^{2} & {\hat{\sigma }}_{02}^{2} \end{array}\right]}^{\;T}$, $W=\left[\begin{array}{l} V_{1}^{T}PV_{1}\\ kV_{2}^{T}V_{2} \end{array}\right]$, $N=N_{1}+N_{2}$, $N_{1}=A^{T}PA$, $N_{2}=kB^{T}B$, $V_{1}=AX-L$, $V_{2}=BX$.

*n*_1_, *n*_2_ are the number of observation equations and constraint equations, respectively. ${\hat{\sigma }}_{01}^{2}$ and ${\hat{\sigma }}_{02}^{2}$ are the variances of unit weight for observation equations and constraint equations, respectively. *V*_1_ and *V*_2_ are the residual vectors for observation equations and constraint equations. Give a small value to *k* as initial weights for constraint equations and the weights for observations are assumed to be unchangeable, ${\hat{\sigma }}_{01}^{2}$ and ${\hat{\sigma }}_{02}^{2}$ can be estimated by Equations (3) and (4) in an iterative manner. During iterations, the *j*th new *k* is reset to:

(5) $$k_{j}=\frac{{\hat{\sigma }}_{01,j-1}^{2}}{{\hat{\sigma }}_{02,j-1}^{2}}k_{j-1}$$

The ${\hat{\sigma }}_{01}^{2}$ and ${\hat{\sigma }}_{02}^{2}$ are repeatedly estimated until 0.999 < ${\hat{\sigma }}_{01}^{2}/\;{\hat{\sigma }}_{02}^{2}$ < 0.001. Note that the initial *k* cannot be too far away from the final *k* as the number of redundant observations is small in this case that the above procedures will have limited capacity to adjust the weights automatically. If the *k* is too large, the iteration may not converge. The values of *k* and variance of united weight estimated by MMSE and HVCE are shown in Table 2. As two methods use different criteria, the estimated *k* and ${\hat{\sigma }}_{0}^{2}$ are different, and hence, different estimated EWHs.

## 4. Equivalent Water Height from GRACE and GLDAS Hydrological Model

In this section, TWS derived from GRACE data and Global Land Data Assimilation System (GLDAS) [51] are used to validate the GPS-derived TSW. Comparisons among the three data sets are conducted.

GRACE is a joint scientific mission of the National Aeronautics and Space Administration (NASA) and the German Aerospace Center that has been measuring the time-variable Earth gravity field since its launch on 17 March 2002 [52]. The degree-60 GRACE Level-2 Release05 (RL05) GSM monthly gravity data product, in the form of spherical harmonic coefficients (SHC), allows us to compute the EWH at a regular grid. These data are accessible solutions calculated from the Center for Space Research (CSR) at University of Texas. The data, spanning between January 2004 and December 2012 are used. Before deriving EWH, the C_20_ term is replaced in the GRACE GSM data by the Satellite Laser Ranging (SLR) results [53]. The degree one terms are added by the results from Swenson et al. [54] to take into account the geocenter motion. To remove spatially correlated errors resulting from the accumulated sum of instrument, orbit, and model errors that generated high uncertainties of stokes coefficients at a higher degree [55], Gaussian smoothing with 350 km averaging radius has been applied [56]. Standard practices are followed to compute EWH. After obtaining the monthly EWH, we use the same temporal model to fit the data as that of GPS. Finally, we obtain the annual amplitudes of the GRACE-derived EWH at 1° × 1° grid.

The Global Land Data Assimilation System (GLDAS) [51], currently has four land surface models: Mosaic, Noah, the Community Land Model (CLM), and the Variable Infiltration Capacity (VIC). The hydrological data used in this study is from the GLDAS-1 Noah model. GLDAS could provide the 3-hourly or monthly 0.25 and 1.0° products globally. In this study, the monthly 0.25° products are employed. In GLDAS model, TWS is the sum of soil moisture in all layers, accumulated snow, and plant canopy surface water, so the groundwater is not included. In Noah model, there are four layers of soil moisture with the maximum depth of 2.0 m, so we sum up the four layers of soil moisture, snow water equivalent, and total canopy water storage to obtain TWS. TWS data from January 2010 to December 2014 are calculated. And then a temporal model consisting of constant, linear and annually varying terms are fitted to the TWS time series at a regular grid. Finally, we obtain the annual amplitude of the TWS from GLDAS-1 Noah model.

## 5. Results and Validation

### 5.1. Results from GPS, GRACE and GLDAS

**Figure 6:**
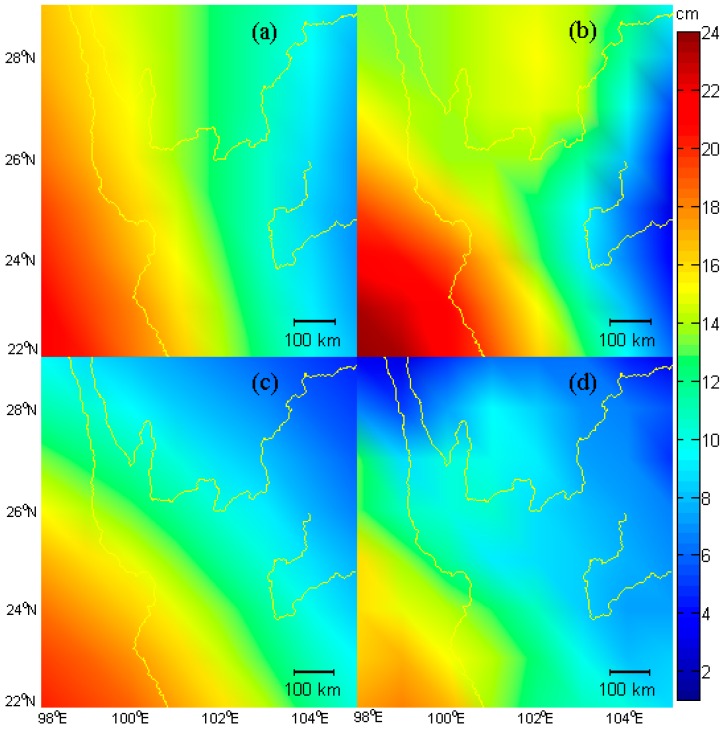
Plots of annual amplitude of EWH in the core areas derived from GPS, GRACE and GLDAS model. (**a**) GPS inferred EWH by HVCE method; (**b**) GPS inferred EWH by MMSE method; (**c**) GRACE derived EWH; (**d**) GLDAS derived EWH.

A surface curve is fitted to the inferred gridded GPS, GRACE and GLDAS EWH results, respectively. To avoid the discrepancies caused by resolution differences, 0.25° × 0.25° GLDAS EWH is smoothed by a 1° × 1° window. Figure 6 shows the plots of the fitted surface curve. The GPS-inferred EWH, by HVCE and MMSE methods, approximately resembles the seasonal water storage change in the study area compared with the GRACE and GLDAS results. The largest seasonal EWH change is observed at the southwest of the research area with a magnitude of ~22 cm and ~25 cm, by HVCE method and MMSE method, respectively, whereas the smallest EWH change is observed at the east of the research area with a magnitude of ~7 cm or ~3 cm, respectively. The GPS-inferred EWHs are larger along the Lancang basin than that in other regions. The whole distribution of the EWH is larger in the west and smaller in the east, and the southwest has the largest EWH change, because of seasonal monsoon around the southeastern Tibet in April/May. This distribution is basically consistent with that of GRACE (Figure 6c) and GLDAS (Figure 6d). The differences between the HVCE method and the MMSE method are that the HVCE method has larger amplitude values in the northwest and smaller values in the north central part than the MMSE method. The MMSE inferred EWH is a little larger than the HVCE EWH in the southwest part and a litter smaller in the east part. The GRACE-inferred EWH presents a decreasing trend of EWH values from southwest to northeast, which is consistent with the GLDAS-inferred EWH. The difference between GPS-inferred EWH and that of GRACE lies in apparently large GPS-inferred EWH in the northern part of the study region.

The EWH within the core area could well be bedetermined, but the EWH in the additional area cannot be determined correctly. According to [46], true/accurate unknowns are necessary to calculate the bias and mean square error which are usually used to assess the precision of ridge estimates. In this case, the EWHs estimated in the additional area are largely inaccurate which greatly affects the precision estimation in the core area. Therefore, we perform a boot-strapping approach to determine the uncertainties of the unknowns from the two methods. During the boot-strapping process, each method is run 29 times with one different station being removed each time, thus generating 29 solutions by each method. We determine the solution uncertainties by calculating the standard deviation of the 29 solutions, which will not be a full error but it gives an indication of the regions sensitive to small data changes.

**Figure 7:**
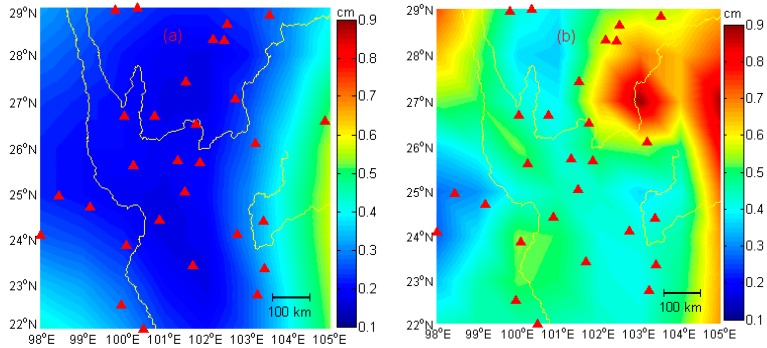
Uncertainties of the two solutions. (**a**) Uncertainties of HVCE method; (**b**) Uncertainties of the MMSE method. The uncertainties are derived by performing a boot-strapping approach.

The resulting uncertainty maps for both the HVCE and the MMSE methods are derived and shown in Figure 7.

In Figure 7a, the uncertainties are very small in most areas except in the east and the southwest parts where few sites are located, with the smallest and the largest EWH lies, respectively (see Figure 6). In Figure 7b, an unevenly distributed uncertainty is obtained when compared to Figure 7a. The uncertainties are large in the east, the northeast and the northwest parts of the area where few GPS sites are located. It can be revealed that much smaller uncertainties can be obtained from the HVCE method when compared to the MMSE method. It also indicates the HVCE method is not as sensitive to the data change as the MMSE method and also may be more reliable.

### 5.2. Quantitative Comparisons among GPS-, GRACE- and GLDAS-Inferred EWH

**Figure 8:**
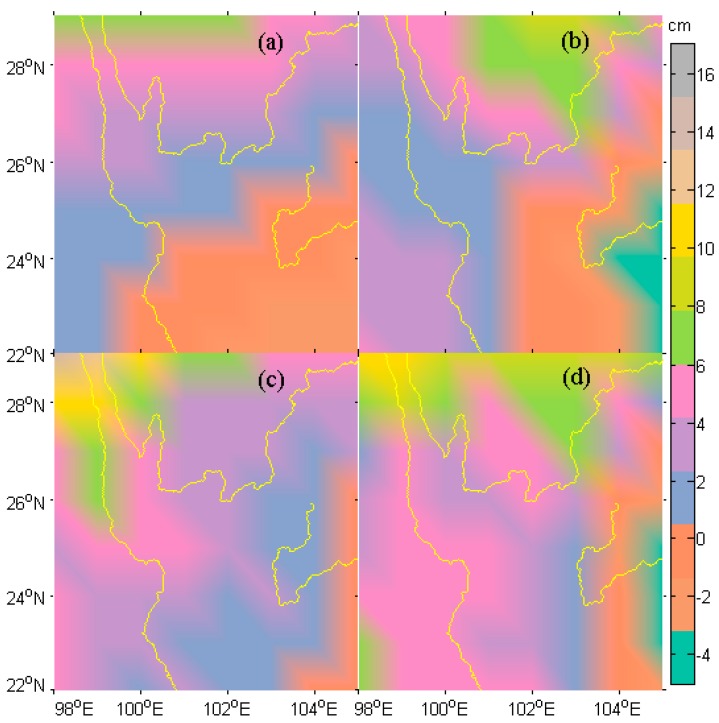
Differences between EWHs derived by different methods. (**a**) GPS(HVCE)-GRACE; (**b**) GPS(MMSE)-GRACE; (**c**) GPS(HVCE)-GLDAS; (**d**) GPS(MMSE)-GLDAS.

The seasonal change in EWH inferred from GPS are compared to that inferred by GRACE and to that predicted by the GLDAS-1 Noah model in a quantitative manner. The difference between the GPS EWH and the GRACE EWH or GLDAS EWH are shown in Figure 8.

The error statistics are shown in Table 3. Overall, the GPS EWH shows in a good agreement with the GRACE EWH, despite the presence of larger discrepancies in the northern part of the study region.

Compared with the GRACE EWH, the GPS EWH computed by HVCE has a bias of 1.9 cm, a standard deviation (SD) of 2.6 cm, and a RMSE of 3.2 cm, with the total of 16.6% larger than the values of GRACE EWH; while the GPS MMSE EWH has a bias of 2.1 cm, a SD of 3.3 cm and a RMSE of 3.9 cm, with the total of 18.0% percent larger than the GRACE EWH. The largest discrepancies mainly happen in the north of the study region. It can be seen from comparison of Figure 8a,b that the HVCE results perform better than the MMSE results, with smaller and smoother discrepancies. Compared with the GLDAS EWH, the GPS HVCE EWH is 39.4% larger and the MMSE EWH is 41.2% larger. The largest discrepancies between the GPS EWH and the GLDAS EWH also occurs in the north. In addition, the EWH estimated by MMSE in the southwest tends to be larger than that estimated by the other methods. In fact, the GPS inferred EWH by [20] in California is about on average 50% larger than the North American Global Land Assimilation System (NLDAS) [57] Noah hydrology model. The GLDAS model or NLDAS model tends to yield a smaller EWH predictions. In this study, the comparison between the GRACE and GLDAS results exhibits the same conclusion, with the GRACE EWH 19.6% larger than that of GLDAS EWH.

### 5.3. Potential Discrepancy and Error Sources

Both the HVCE method and MMSE method qualitatively resolve the seasonal TWS changes from GPS observations, when compared to GRACE-inferred one, however, yielding slightly different results. The HVCE method estimates a larger regularization parameter than the MMSE method, hence, giving a smoother EWH. The discrepancy in HVCE result manifested in the northwest (Figure 8a), probably due to the absence of GPS stations in this sub-region. The discrepancy in MMSE result revealed in the north (Figure 8b), probably due to the larger estimation of GPS annual vertical oscillations. The amplitude at station SCSM is 6.98 mm, which is more than 1 mm (corresponds to ~2 cm EWH) larger than those at the nearby stations (i.e., SCYX, SCMB, SCMN) (see Figure 4). This causes the MMSE EWH estimation in this area to be larger when the regularization parameter is small. Overall, the poor spatial distribution and quality of the GPS observations does not well constrain the EWH in the north of the study area.

As the GLDAS TWS is the sum of soil moisture in all layers, accumulated snow, and plant canopy surface water while the GPS- or GRACE-inferred EWH is the total TWS, including water in reservoirs and rivers, snow on the ground, water in the soil, even water under the ground. There are a lot of rivers and reservoirs in the study area. The water changes in these rivers and reservoirs are not fully considered in the GLDAS model but these changes can be potentially detected by GRACE or GPS, which will cause the GLDAS predicted EWH to be smaller than the GPS or GRACE inferred EWH. From another perspective, this difference between the GPS (GRACE) and GLDAS model may help us infer the water storage change in the rivers and reservoirs, and hence, providing a further constraint to the hydrology models. If this can be achievable, GRACE can more accurately determine the sub-surface waters in the upper layer by removing the GLDAS TWS and water storage in rivers and reservoirs from the total TWS inferred by GRACE.

However, as a potential technique to detect seasonal TWS change, GPS suffers some error sources that can hardly be removed. A period of ~351.4 days is needed for the Sun to return to the same point in space relative to the GPS orbital nodes (as viewed from the Earth), which introduces the so-called GPS draconitic error with a period of 351.2 ± 2.8 days [58,59]. So far, this error cannot be separated from the mass change induced position changes since the time spans of available GPS time series are not long enough. In addition, the land surface temperature variation will cause the thermal elastic deformations. According to the analytical results from Fang et al. [60], the thermally induced displacement can be as large as ~2 mm in the radial component and ~1 mm in the transverse component. These two effects are irrelevant to surface mass change but can be detected by GPS, which will certainly contaminate the annual amplitude associated with TWS change. These are the disadvantages of GPS relative to the GRACE in seasonal TWS change detection.

## 6. Conclusions

Recent advances in the usage of GPS for potential extraction of equivalent water height have been demonstrated in the California region [20]. In this study, we first use the GPS vertical observations to infer the seasonal water change in the Lancang River, the Yangtze River, and the Zhujiang River basins, making up for the absence of hydrological data. The seasonal equivalent water height (EWH) change in this region are first resolved with 1° × 1° resolution using 29 GPS stations. The largest EWH change is observed at the southwest corner of research area with a magnitude of ~22 cm (HVCE) or ~25 cm (MMSE) corresponding to a seasonal vertical motion of ~9 mm. The smallest is at the east central part with a magnitude of ~7 cm (HVCE) or ~3.0 cm (MMSE) corresponding to a seasonal vertical motion of ~3 mm. Between HVCE and MMSE methods, HVCE method gives a slightly better results than the MMSE method. As no published hydrological data are available in this study region, the GPS inferred seasonal EWH change are compared by GRACE results and GLDAS model. The GPS inferred EWH is about 16.6%–18.0% larger than the GRACE inferred EWH and is about 39.4%–41.2% larger than the GLDAS inferred EWH. This larger discrepancy between GPS and GLDAS may be potentially due to the impact of water storage change in rivers and reservoirs. The discrepancy between GPS and GRACE may be due to the different sensitivities to the surface mass change. Last but not least, the GPS draconitic error and the thermal elastic deformations that share a similar period with the annual TWS signal, will certainly contribute to this discrepancy.

## Figures and Tables

**Table 1:** White noise, Power law noise, spectral index, and annual amplitude estimates for the vertical component of the 29 GPS time series, and the power law noise is scaled to mm/year^1/4^ for comparison’s sake. As Hector program produces unreliable results at SCNN sites when the spectral index is not fixed, so does another program CATS V3.1.2 [40], so the parameters and noise at SCNN are estimated with fixed spectral index −1.

Site	White Noise, mm	Power Law Noise, mm/year^1/4^	Sepctra Index	Amplitude, mm
GZSC	2.00	13.21	−0.54 ± 0.23	3.03 ± 0.43
SCMB	2.45	11.49	−0.67 ± 0.23	6.08 ± 0.51
SCMN	2.63	10.35	−0.66 ± 0.21	5.93 ± 0.46
SCNN	4.75	9.80	−1.00 ± 0.00	8.21 ± 0.95
SCPZ	2.91	7.21	−0.77 ± 0.23	6.67 ± 0.41
SCSM	3.69	7.53	−0.98 ± 0.24	6.98 ± 0.64
SCXC	2.64	6.12	−1.10 ± 0.18	6.27 ± 0.66
SCXD	3.38	3.55	−1.34 ± 0.25	6.39 ± 0.64
SCYX	3.48	7.96	−0.97 ± 0.23	4.65 ± 0.66
SCYY	3.06	3.16	−1.24 ± 0.27	6.19 ± 0.47
XIAG	3.02	9.96	−0.94 ± 0.11	5.66 ± 0.54
YNCX	3.07	8.90	−0.78 ± 0.19	5.40 ± 0.44
YNDC	4.64	12.85	−0.60 ± 0.26	5.66 ± 0.46
YNJD	4.23	14.46	−0.69 ± 0.21	8.10 ± 0.58
YNJP	4.08	8.70	−1.09 ± 0.16	5.63 ± 0.78
YNLA	4.36	9.34	−1.09 ± 0.17	8.82 ± 0.84
YNLC	4.43	9.08	−0.82 ± 0.27	8.84 ± 0.51
YNLJ	2.52	5.90	−0.95 ± 0.17	6.41 ± 0.40
YNMH	4.05	3.52	−1.44 ± 0.23	7.72 ± 0.67
YNMJ	3.00	13.18	−0.57 ± 0.20	7.30 ± 0.42
YNML	3.51	7.03	−0.68 ± 0.31	3.87 ± 0.31
YNMZ	4.00	16.38	−0.64 ± 0.19	6.57 ± 0.60
YNRL	3.96	3.53	−1.21 ± 0.30	8.50 ± 0.43
YNSD	3.18	12.72	−0.76 ± 0.17	8.17 ± 0.58
YNTC	3.68	14.69	−0.68 ± 0.21	8.81 ± 0.57
YNTH	3.43	9.24	−0.82 ± 0.19	4.77 ± 0.49
YNYA	3.43	4.82	−1.14 ± 0.22	6.18 ± 0.49
YNYM	3.36	10.19	−0.77 ± 0.19	6.81 ± 0.48
YNYS	3.01	9.93	−0.55 ± 0.25	7.46 ± 0.32
Mean	3.28	9.60	−0.86	6.59

**Table 2:** Values of *k* and ${\hat{\sigma }}_{0}^{2}$ estimated by MMSE and HVCE.

Method	*k*	${\hat{\sigma }}_{0}^{2}$ (mm^2^)
MMSE	1.95 × 10^−5^	0.86
HVCE	1.99 × 10^−4^	1.05

**Table 3:** Statistics of the differences between EWHs, and the Percentage Errors are calculated by Bias (a–b)/mean (b).

	Bias (cm)	SD (cm)	RMSE (cm)	Percentage Error (%)
GPS_HVCE-GRACE	1.9	2.6	3.2	16.6
GPS_MMSE-GRACE	2.1	3.3	3.9	18.0
GPS_HVCE-GLDAS	3.8	3.0	4.8	39.4
GPS_MMSE-GLDAS	4.0	3.4	5.2	41.2
GPS_HVCE-GPS_MMSE	-0.2	2.0	2.0	-1.3
GRACE-GLDAS	1.9	1.6	2.5	19.6
